# Supplementary figures and images for: Seasonal tissue-specific gene expression in wild crown-of-thorns starfish reveals reproductive and stress-related transcriptional systems
Source: PLoS Biol. 2024 May 14;22(5):e3002620. doi: 10.1371/journal.pbio.3002620 (PMC11093393; doi:10.1371/journal.pbio.3002620)

S1 Figure

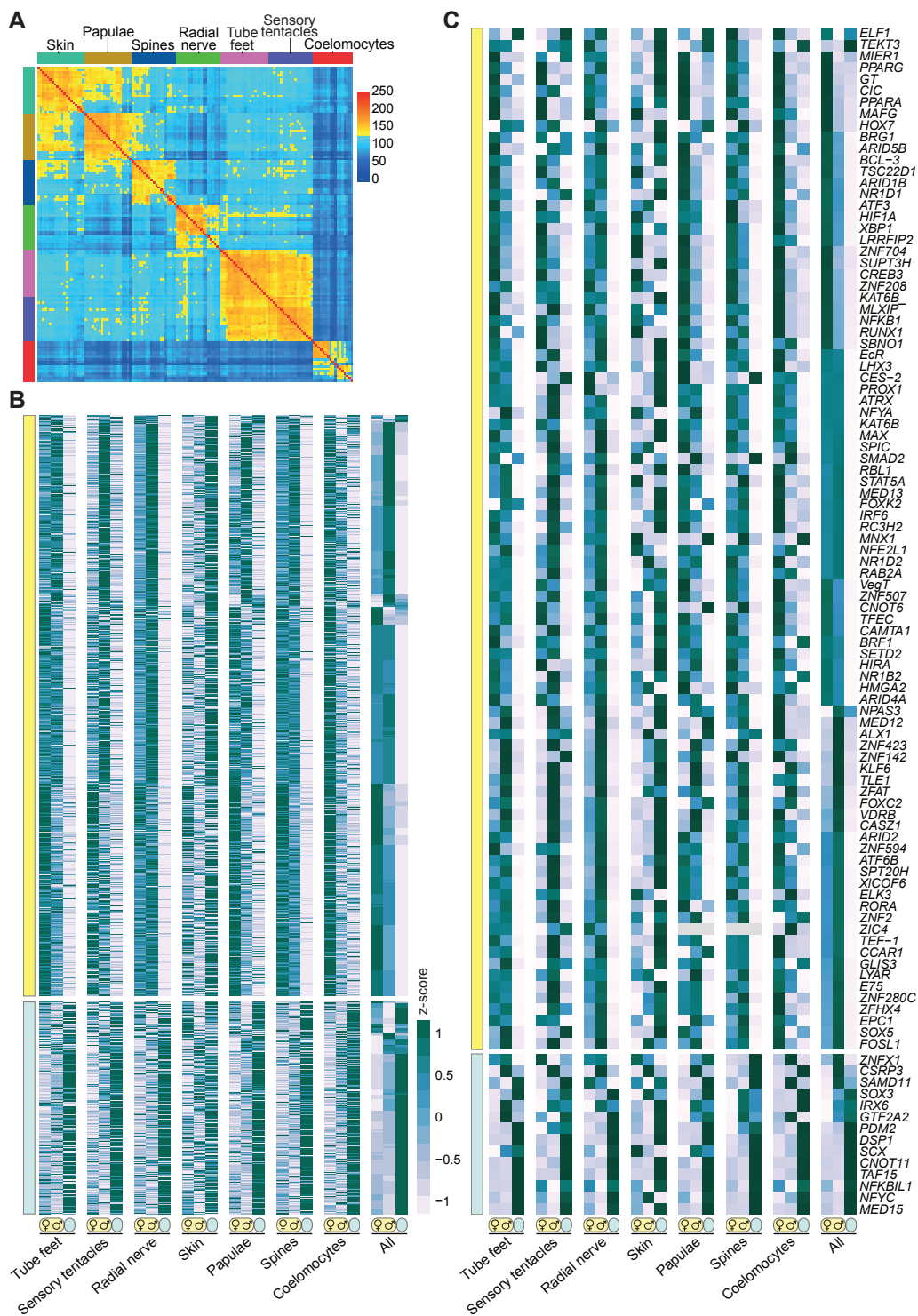

Supplement: S1 Fig — (A) Hierarchical clustered heatmap of Pearson correlation of expressed protein-coding genes across the 7 tissue transcriptomes. (B) Heatmap of the 2,079 protein-coding genes that are significantly differentially expressed between seasons in at least 1 tissue (adjusted p-value <0.05). The genes up-regulated in the summer (yellow) and winter (blue) are at the top and bottom of the heatmap, respectively. The “All” heatmap shows the average expression across all tissues across for both sexes and seasons. (C) Heatmap of the TFs that are significantly differentially expressed COTS tissues (adjusted p-value <0.05). The data underlying this figure can be found in S3 and S5 Tables and at https://doi.org/10.5281/zenodo.10831187. (PDF) [file pbio.3002620.s001.pdf]

S2 Figure

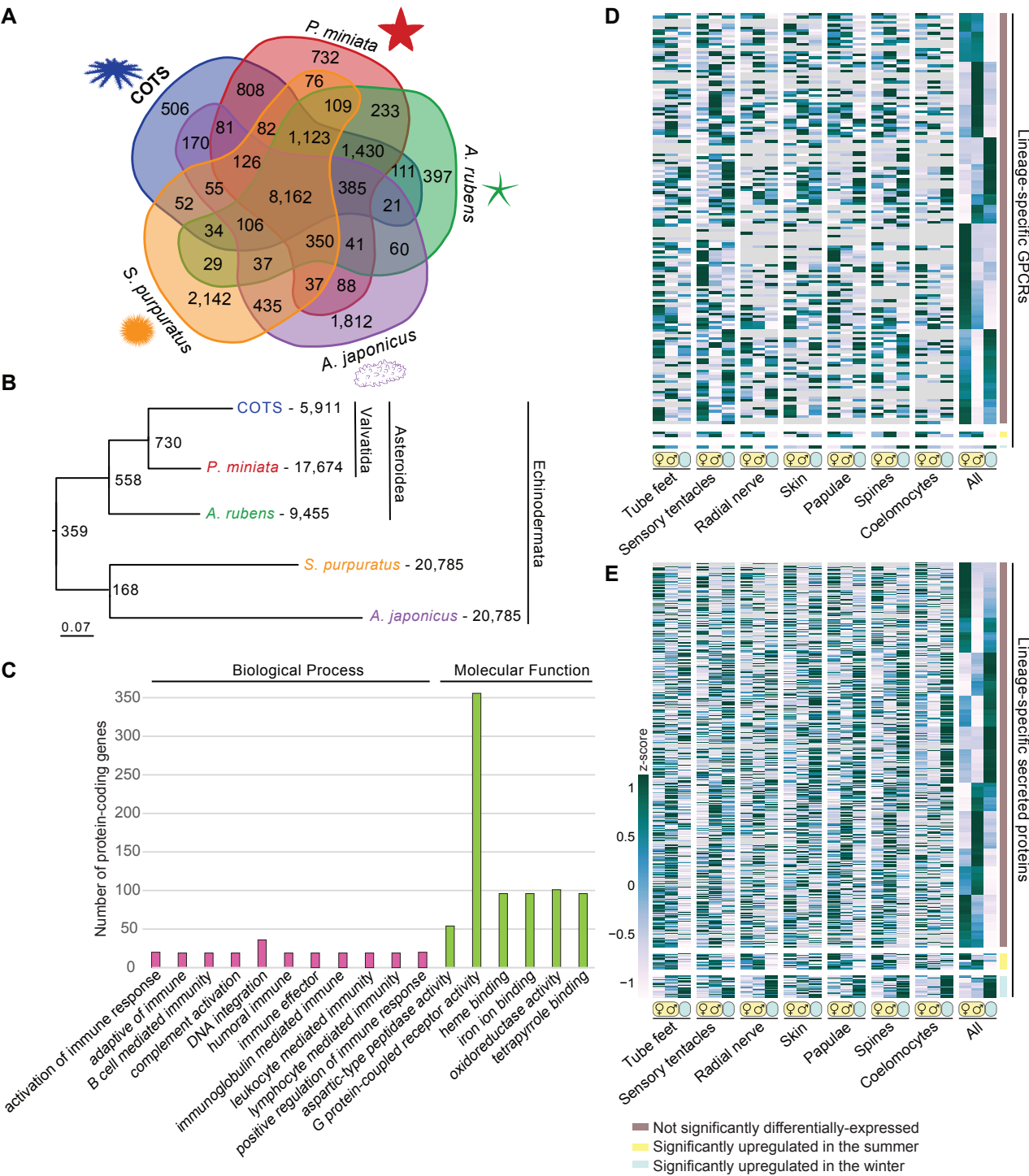

Supplement: S2 Fig — (A) Venn diagram showing the overlap of orthologous gene groups among 5 echinoderms, the sea urchin Strongylocentrotus purpuratus [65], the sea cucumber Apostichopus japonicus [66], and starfish Asterias rubens, Patiria miniata [67], and Acanthaster cf. solaris (COTS) [21,29]. COTS has 506 unique gene groups. (B) Phylogenetic tree of the 5 echinoderms showing gene duplication events at each node and branch. The taxonomic ranks are listed on the right. The numbers next to each species name are the total number of lineage-specific gene duplication events as detected by Orthofinder. (C) GO term enrichments of COTS-specific genes. The x-axis shows the GO terms and the y-axis shows the number of protein-coding genes in each term. (D, E) Expression profiles of lineage-specific GPCRs and secreted proteins in wild COTS. The heatmaps show the scaled expression levels (z-score) based on TPM normalized reads for each tissue in 7 females, 6 males, and 7 winter individuals. The “All” heatmap shows the average expression across all groups and tissues. The heatmaps are colour-coded (right) by significant differential expression (DESeq2; adjusted p-value <0.05): not significantly differentially expressed (brown), significantly up-regulated in the summer (yellow) and significantly up-regulated in the winter (blue). The data underlying this figure can be found in S7 Table and at https://doi.org/10.5281/zenodo.10831187. (PDF) [file pbio.3002620.s002.pdf]

## S3 Figure

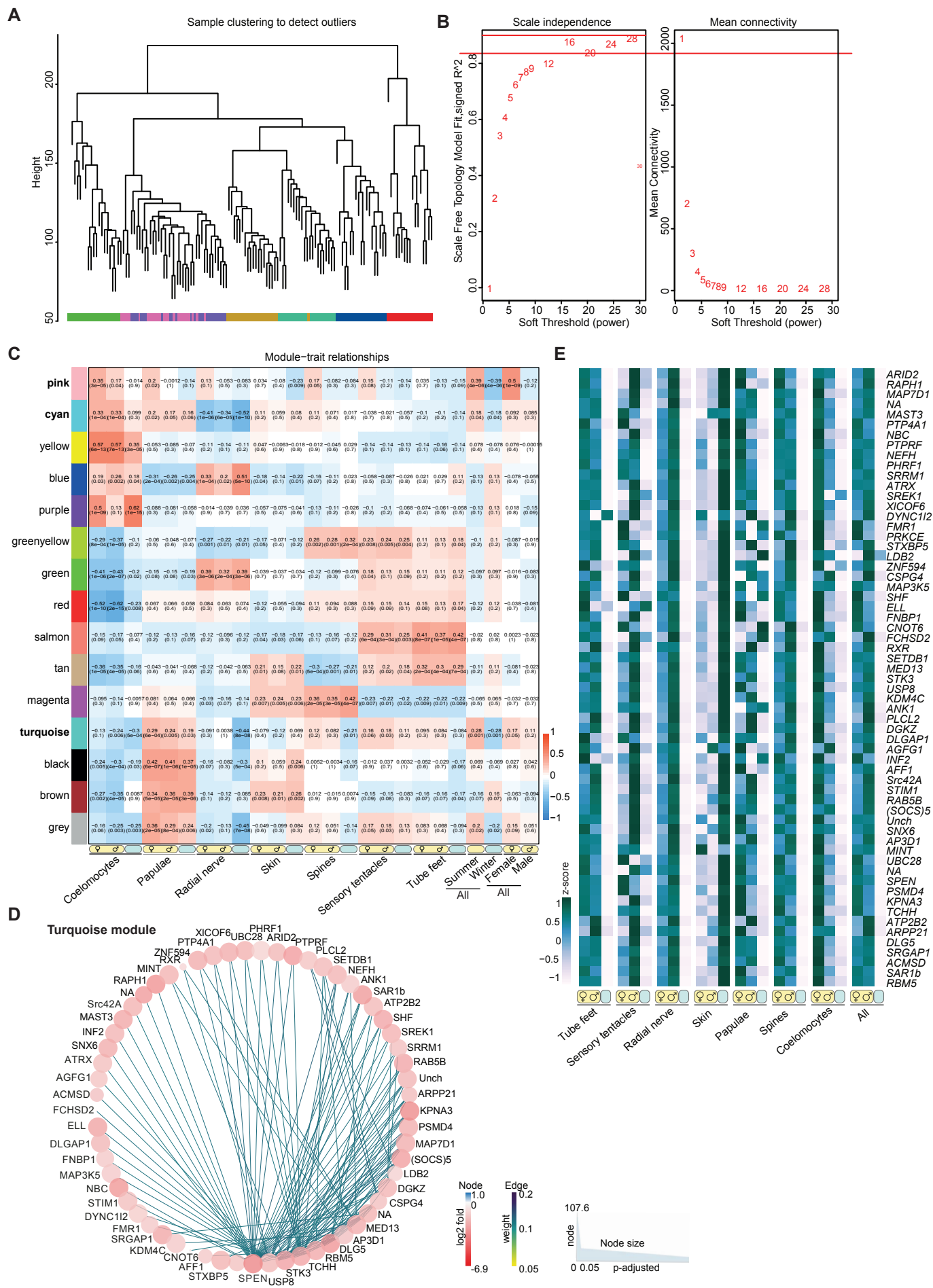

Supplement: S3 Fig — (A) Clustering dendrogram of all samples. The colour bar at the bottom corresponds to COTS tissues: green, radial nerve; pink, tube feet; purple, sensory tentacles; brown, papulae; turquoise, skin; blue, spines; red, coelomocytes. (B) Scale-free topology model fit for different soft-thresholding powers (left). The signed R2 value measures how well the scale-free topology assumption fits the observed network connectivity distribution. Mean network connectivity for different soft-thresholding powers (right). The mean connectivity is the average number of connections (edges) per node (gene) in the network for a given power value. (C) Module-trait associations. Each column represents a trait (tissue/season/sex), and each row represents an identified module. Red and blue colour notes positive and negative correlation with the trait, respectively. Numbers in each cell represent the correlation coefficient and the p-value (in brackets). (D) Gene interaction networks for the turquoise module (edge weights >0.164). The size of each coding sequence (node) in the network corresponds to the significance of differential expression (up-regulated in the summer; p-adjusted value in DESeq2). The colour corresponds to the log2fold change in gene expression: red, up-regulated in summer; blue, up-regulated in winter. (E) Expression profiles of the core hub genes in the turquoise module. This heatmap shows the scaled expression levels (z-score) based on TPM normalised reads for each tissue in females, males, and winter individuals. The “All” heatmap shows the average expression across all groups and tissues. The data underlying this figure can be found in S8 Table and at https://doi.org/10.5281/zenodo.10831187. (PDF) [file pbio.3002620.s003.pdf]

S4 Figure

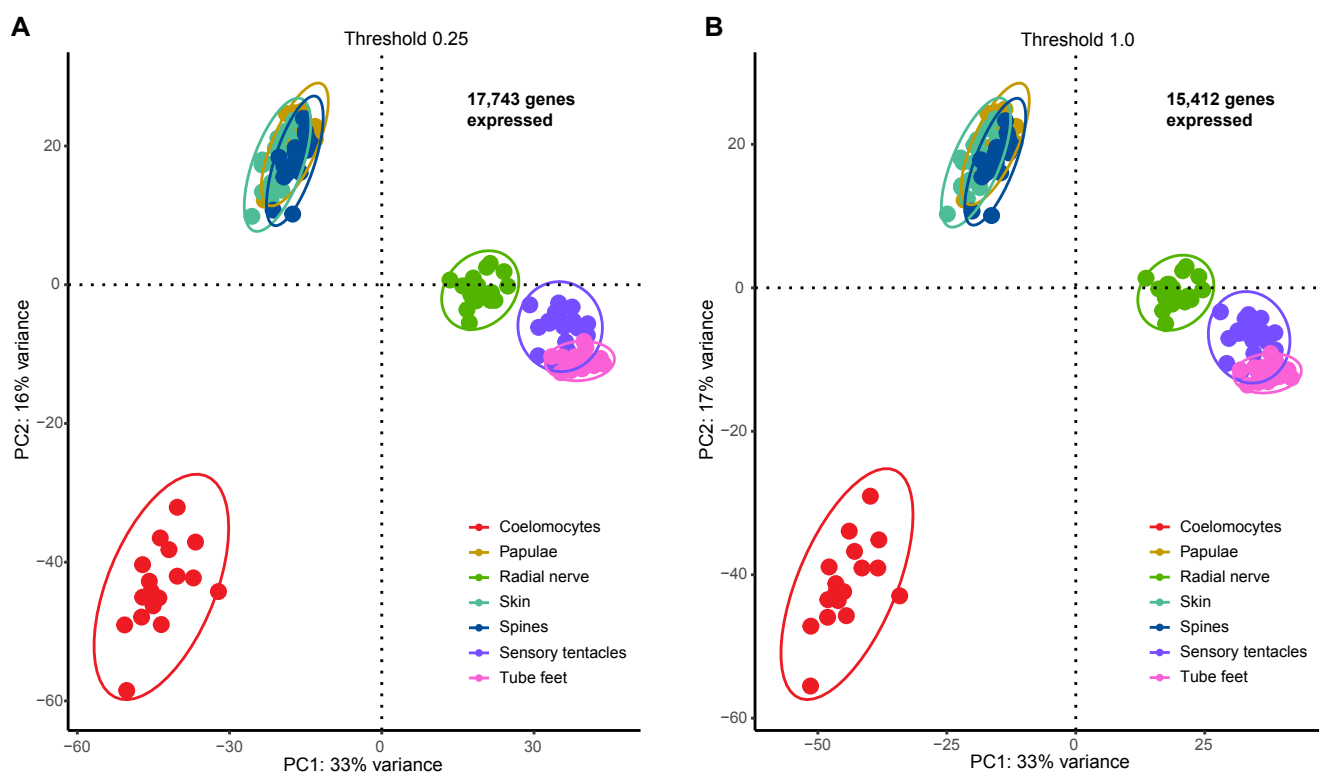

Supplement: S4 Fig — PCA of tissue transcriptomes with an average expression threshold ≥0.25 (A) and ≥1 (B); 95% confidence ellipses shown. Pink, tube feet; purple, sensory tentacles; green, radial nerve; turquoise, skin; brown, papulae; blue, spines; red, coelomocytes. (PDF) [file pbio.3002620.s004.pdf]
