# Supplementary material for: Seasonal tissue-specific gene expression in wild crown-of-thorns starfish reveals reproductive and stress-related transcriptional systems
Source: PLoS Biol. 2024 May 14;22(5):e3002620. doi: 10.1371/journal.pbio.3002620 (PMC11093393; doi:10.1371/journal.pbio.3002620)
Supplement: Attachment — Submitted filename: Response to Reviewers.pdf [file pbio.3002620.s013.pdf]

Seasonal tissue-specific gene expression reveals reproductive and stress-related transcriptional systems in wild crown-of-thorns starfish

M. Morin, M. Jönsson et al.

We thank you - *PLOS Biology* editors, Academic Editor with relevant expertise and three independent reviewers – for assessing this manuscript, and appreciate your efforts. We are pleased by your overall positive response to the manuscript, and the constructive nature of your comments and suggestions.

Editorial recommendations and three scientific assessments are listed below in bold. Our responses to each comment are in plain text and prefixed by "Response:".

<u>**Editor's summary assessment**</u>

Reviewer #1 is positive, having no actual requests, but regretting the lack of gonadal data and validation by qPCR and/or FISH (we will not require these). Reviewer #2 is also positive, but has constructive suggestions to improve the contextualisation in the Intro and the dataviz (converting Table 2 into a heatmap; reformatting the heatmaps in Figs 2 and 3), and wonders if you could help readers interpret the WGCNA results more easily. Reviewer #3 is positive and has very minor requests.

In light of the reviews, which you will find at the end of this email, we are pleased to offer you the opportunity to address the comments from the reviewers in a revision that we anticipate should not take you very long. We will then assess your revised manuscript and your response to the reviewers' comments with our Academic Editor aiming to avoid further rounds of peer-review, although might need to consult with the reviewers, depending on the nature of the revisions.

Response: We are pleased by the overall positive assessment and will systematically address editorial recommendations and individual reviewer comments below.

IMPORTANT:

a) Please address the concerns raised by the reviewers; in particular, we think that the presentational suggestions from reviewer #2 will help the accessibility and appeal of your paper.

Response: We have revised text and figures as recommended by reviewer #2. The details regarding these changes are outlined below under reviewer #2's comments.

b) Please comply with our Data Policy; specifically, we need you to supply the numerical values underlying Figs 1BCDE, 2ABCDE, 3ABCDEFG, S1CDE, S2ABC, S3AB, either as a supplementary data file or as a permanent DOI'd deposition.

Response: All data underlying these figures are included in Supporting Information (S1-S8 Tables). We have added an additional supplementary tables (S7 Table and several tabs in the existing S Tables) to the Supporting Information, two large data files have been deposited in the public repository Zenodo, and scripts and input and output files deposited in github. These URLs are now cited appropriately in the Figure legends and Materials and Methods.

c) Please cite the location of these data clearly in all relevant main and supplementary Figure legends, e.g. "The data underlying this Figure can be found in S1 Data" or "The data underlying this Figure can be found in https://doi.org/10.5281/zenodo.XXXXX"

Response: We have inserted this information throughout the main text and figure legends, including supplementary figure legends. All Supporting Information files are included with the submission, except two large speadsheets, which are now publicly available (https://doi.org/10.5281/zenodo.10831187).

d) Please make any custom code available, either as a supplementary file or as part of your data deposition.

Response: Scripts used of DESeq2, WGCNA and GO enrichments, along with input and output files are publicly available (https://github.com/MathiasJoensson/Supplementary-data-COTS-seasonal-tissue-transcriptomes) and cited in the Materials and Methods.

****IMPORTANT - SUBMITTING YOUR REVISION****

Your revisions should address the specific points made by each reviewer. Please submit the following files along with your revised manuscript:

1. A 'Response to Reviewers' file - this should detail your responses to the editorial requests, present a point-by-point response to all of the reviewers' comments, and indicate the changes made to the manuscript.
***NOTE: In your point-by-point response to the reviewers, please provide the full context of each review. Do not selectively quote paragraphs or sentences to reply to. The entire set of reviewer comments should be present in full and each specific point should be responded to individually.**

Response: This 'Response to Reviewers' file provides a point-by-point response to all editorial and reviewer comments.

You should also cite any additional relevant literature that has been published since the original submission and mention any additional citations in your response.

Response: We have updated the references including relevant articles that have been published since the original submission. These can be viewed in the "Revised Article with Changes Highlighted" file.

2. In addition to a clean copy of the manuscript, please also upload a 'track-changes' version of your manuscript that specifies the edits made. This should be uploaded as a "Revised Article with Changes Highlighted " file type.

Response: In our resubmission we have included both a clean copy and an annotated version named "Revised Article with Changes Highlighted ".

Resubmission Checklist
When you are ready to resubmit your revised manuscript, please refer to this resubmission checklist: https://plos.io/Biology_Checklist

Response: We have followed this checklist.

To submit a revised version of your manuscript, please go to https://www.editorialmanager.com/pbiology/ and log in as an Author. Click the link labelled 'Submissions Needing Revision' where you will find your submission record.

Please make sure to read the following important policies and guidelines while preparing your revision:

Published Peer Review
Please note while forming your response, if your article is accepted, you may have the opportunity to make the peer review history publicly available. The record will include editor decision letters (with reviews) and your responses to reviewer comments. If eligible, we will contact you to opt in or out. Please see here for more details:
https://blogs.plos.org/plos/2019/05/plos-journals-now-open-for-published-peer-review/

PLOS Data Policy
Please note that as a condition of publication PLOS' data policy (http://journals.plos.org/plosbiology/s/data-availability) requires that you make available all data used to draw the conclusions arrived at in your manuscript. If you have not already done so, you must include any data used in your manuscript either in appropriate repositories, within the body of the manuscript, or as supporting information (N.B. this includes any numerical values that were used to generate graphs, histograms etc.). For an example see here: http://www.plosbiology.org/article/info%3Adoi%2F10.1371%2Fjournal.pbio.1001908#s5

Response: All data generated and used in this study can be found in the body of the manuscript, Supporting Information and in public repositories. In addition to the tables included in the Supporting Information, (i) two large data files with filtered read counts and normalised TPMs, and DESeq2 statistics have been uploaded to public repository (https://doi.org/10.5281/zenodo.10831187) , (ii) scripts used of DESeq2, WGCNA and GO enrichments, along with input and output files are publicly available (https://github.com/MathiasJoensson/Supplementary-data-COTS-seasonal-tissue-transcriptomes) (iii) the raw RNA sequences generated in this study are publicly available in NCBI Sequence Read Archive (SRA) under BioProject PRJNA821257, and (iv) the new GBR v1.1 gene models and transcriptomes generated can be visualized at: https://apollo-portal.genome.edu.au/degnan/cots. We have added a statement to Materials and Methods to alert readers to these resources.

Protocols deposition
To enhance the reproducibility of your results, we recommend that if applicable you deposit your laboratory protocols in protocols.io, where a protocol can be assigned its own identifier (DOI) such that it can be cited independently in the future. Additionally, PLOS ONE offers an option for publishing peer-reviewed Lab Protocol articles, which describe protocols hosted on protocols.io. Read more information on sharing protocols at https://plos.org/protocols?utm_medium=editorial-email&utm_source=authorletters&utm_campaign=protocols

Response: The data generated in this manuscript uses established methods, thus there is no need to deposit detailed protocols. The Materials and Methods section alerts readers to previous publications and other resources. We have made all customised scripts and input files available in github (https://github.com/MathiasJoensson/Supplementary-data-COTS-seasonal-tissue-transcriptomes). These are cited in the Materials and Methods

Thank you again for your submission to our journal. We hope that our editorial process has been constructive thus far, and we welcome your feedback at any time. Please don't hesitate to contact us if you have any questions or comments.

Reviewer #1

Summary
Wildtype animals in their native habitats, are often overlooked when considering experimental models. Most animals though, do not live in laboratories, housed under constant conditions, with ample food, conditioned light, and clean conditions. Biology is impacted by changes in the world, by reproduction, temperature change and physical conditions that constantly vary. The current dataset is a very nice demonstration of such dynamic changes in an important animal model.

The data are RNA-seq experiments of different tissues from different animals from different times of the year. As such - it is exactly what investigators need in order to understand biology. These datasets will be very useful in looking at any further analysis of especially marine organisms.

Response: Thank you for your overall positive assessment of our approach and the manuscript.

Critique
It is unfortunate that the investigators did not include gonads in their analysis. Although gonads have been sequenced from this animal, they would serve as an excellent and essential foundation for measuring changes in other somatic tissues. Without the gonads, we are missing a key baseline for which all other tissues can be compared. It would serve as a seasonal control, especially since in this animal, the reproductive cycles are so well known. Further, the gonads may well be the regulators of all the other tissues sampled and we are left guessing what correlative changes we might find were those data also present.

Response: We would have liked to have included gonads in this study, however the mode of sampling (immediately upon collection on marine vessels at sea) and the sampling regime (small and precise tissue biopsies) precluded us procuring these in the winter, when the gonads were absent or reduced to an extent that they were not visually discernible from the underlying viscera.

Although we were not able to undertake a seasonal comparison of gonad gene expression, we previously have demonstrated that large differences exist between female and male gonads and somatic tissues prior to a spawning event (Jonsson et al. 2022. BMC Biol 20: 288). The seasonal analysis of the latter in the current manuscript has revealed sexual dimorphic gene regulation likely underlies differences in gene expression in fecund female and male COTS (new Fig 2D and S5 Table).

Further, as valuable as the datasets are - it would be important to have some supporting data to test RNA-values by e.g. qPCR, and to have in situ RNA hybridizations to see which cells of the various tissues the genes are actually expressed. These correlative experiments would help the reader better understand context for within the space that the data must sit.

Response: Thank you for these suggestions. Our sampling regime – multiple biological replicates for each somatic tissue in each season and sex (new Fig 1D,E, S2-4 Tables and https://doi.org/10.5281/zenodo.10831187) – provides a statistical perspective on gene expression that can not be readily achieved by in situ hybridization or quantitative RT-PCR analyses on individual starfish. Our dataset accounts for gene expression variation between individuals and seasons. We elected to emphasise genes that we deem significantly differentially expressed using Wald Test statistics in the DESeq2 package with an adjusted *p*-value < 0.05 between conditions (tissues, seasons and sex). This is a more rigourous approach compared to qRT-PCR, which is reliant on high levels of similarity of reference gene expression between individuals. Thus, we feel that both in situ hybridization and qRT-PCR will not further improve the conclusions drawn from the replicated transcriptomic data used in the current study.

Overall conclusion
Valuable datasets are given and will be useful by the wider community. The impact will however be limited by the absence of key foundational tissue measurements and follow-up examples.

Response: Yes, further analyses would shed light of the molecular and cell biology of seasonal changes in COTS tissues. However, these are ancillary to the primary aim of this study and would not add to the current conclusions based on transcriptomics.

Reviewer #2

In this manuscript, the authors thoroughly analyze tissue transcriptomes from the crown-of-thorns starfish (COTS) across the summer and winter seasons. Investigating seven major tissue types revealed seasonal and sex-specific variations in gene expression. Despite its complexity, the employment of weighted gene correlation network analysis (WGCNA) for identifying co-expressed genes effectively highlights the summer upregulation of various signaling and transcription factors, mainly in females.

Nevertheless, while valuable, the extensive tissue data appears to have detracted from a clear presentation of seasonal differences. Although the manuscript is competently written and features adequate visual representation, refinements in phrasing and data visualization could significantly improve its publication readiness and accessibility to a broader audience.

Response: We thank you for these critical comments. We agree that presenting such a large dataset is challenging. As recommended, we have revised the text and figures to hopefully improve readability. These changes are outlined below.

Major comments:

1. The introduction could be expanded to give readers a better understanding of the topic, especially regarding seasonal expression changes in other animals.

Response: We have added a sentence to the end of the Introduction about this. To our knowledge, there are few comparable studies of seasonal gene expression using transcriptomics in animals, and none in marine animals; a human study was published in *PLOS Biology* in 2023, which is cited in this manuscript. Thus, there is little further background that we can add on this subject.

2. Presenting tissue-specific seasonal changes is currently cumbersome; for instance, Table 2 is challenging to interpret and offers limited experimental insight. Converting this into a heatmap integrated into Fig. 2 might enhance readability.

3. Patterns like those in Fig. 2A and B are more discernible. Reformating heatmaps in Figs. 2 and 3 could make seasonal and sex-specific expression patterns more apparent, highlighting areas of co-expressed genes with significant upregulation or downregulation.

Response: As recommended, we have converted the results in Table 2 into a bar graph (new Fig 2A) and deleted the table. Following on from this general recommendation, we also converted Table 1 into a figure (new Fig 1C). In revising Fig 2 we moved the hierarchical clustered heatmap of Pearson correlation of expressed protein-coding genes across the seven tissue transcriptomes (old Fig 2D) into Supporting information (new S1A Fig) as it provided similar information to the PCA (new Fig 1D). We also translocated the transcription factor heat map into S1C Fig in its place we added a summary bar graph similar to the new Fig 2A (new Fig 2E) and simplified Fig 2F. We also added a

new panel (new S1B Fig) to the Supporting Information, which displays as a heat map all significantly differentially expressed genes in winter and summer.

4. While using WGCNA to identify co-expressed genes is logical, interpreting these results is complex. Could statistical analysis pinpoint season-specific genes and categorize them by tissue specificity? Alternatively, focusing only on seasonal and sex differences in Fig. 3A might simplify the analysis.

Response: We have taken this advice and revised Fig 3 and accompanying new S3 Fig (old S2Fig), focusing on the pink module (stress genes uniquely upregulated in gravid females) in the main figure and moving the turquoise module (upregulated in the summer) to S3 Fig. These changes simplify the graphical presentation of the results.

5. The manuscript uses interchangeable terms for 'protein-coding gene,' such as protein-coding mRNAs, coding sequences, and protein-coding sequences. A uniform terminology is recommended.

Response: As recommended, in the revision we have consolidated these terms into "protein-coding genes".

Minor comments:

6. "Like most marine animals, ..." could be more precisely directed at marine invertebrates to avoid confusion with marine mammals.

Response: We have selectively changed "marine animals" to "marine invertebrates", as appropriate.

7. The color coding for cyan and turquoise modules is too similar, making them hard to differentiate.

Response: With the revision of Fig 3 and the movement of the turquoise graphs into Supporting Information, we feel that this is no longer necessary. The written names clearly discriminate these two modules.

8. Adding line numbers would facilitate more accessible manuscript review.

Response: Agree – our apologies.

<u>Reviewer #3</u>

This work by Morin et al. provides interesting datasets about the physiological ecology of the wild crown-of-thorns starfish by the tissue-, season- and sex-level broad transcriptomic analysis. Although the method of this study seems similar to those of previous research including previous works of the authors themselves, the Cell-Seq2 transcriptomic datasets of the COTS caught in different seasons will be the good references to study about the annual reproductive cycle, and other year-round behaviors and ecologies of the echinoderms. Handling of the datasets in this study seems good, and the discussion for each result is mostly reasonable. Following are some minor points to be collected and/or explained.

Response: Thank you for your positive and constructive comments.

Materials and Methods

* **Section 2, Paragraph 2: Is the reference genome of Apostichopus japonicus downloaded by NCBI? If it is true, please adjust the place of parentheses to avoid ambiguity.**

Response: We corrected and reorganised this sentence to hopefully improve readability.

* **Section 3, Paragraph 2: Please describe what does "VST" mean in this paragraph instead of S4, P2.**

Response: They both mean the same thing (variance-stabilising transformed). We have explained “VST” when it first appears in the text.

Fig 1A:

* **Please describe the value of the scale bar.**

Response: We have revised the figure so that the longitudes and latitudes are now presented. Thus there is no need for a scale bar in this figure and it has been removed.

S1 Fig. B:

* **What the numbers on the right of each species' name mean?**

Response: The numbers next to each species name represent the total number of lineage-specific gene duplication events as detected by Orthofinder. We have revised the legend (this in now S2 Fig), and also have created new tables within the S7 Table file to provide more general Orthofinder results.

* **It would be better to refer how this result (gene duplication events at each node and branch) is related to the authors' discussion in the main manuscript.**

Response: We state this link in the Results and have added a sentence to the Discussion that highlights the link between release of species-specific proteins into the surrounding sea water and differential gene expression in the spines
